# Correlates of smoking among youth: the role of parents, friends, attitudes/beliefs, and demographics

**DOI:** 10.1186/s12971-016-0072-0

**Published:** 2016-03-24

**Authors:** Noella A. Dietz, Kristopher L. Arheart, David F. Sly, David J. Lee, Laura A. McClure

**Affiliations:** Department of Public Health Sciences, Sylvester Comprehensive Cancer Center, University of Miami Miller School of Medicine, 1120 NW 14th Street, 9th Floor C202, Miami, FL 33136 USA; Department of Public Health Sciences, University of Miami Miller School of Medicine, 1120 NW 14th Street, 10th Floor, Miami, FL 33136 USA; Florida State University, College of Social Sciences, 1860 Rabalo Drive #106A, Vero Beach, FL 32960 USA; University of Miami Miller School of Medicine, Sylvester Comprehensive Cancer Center, 1120 NW 14th Street, 9th Floor, Miami, FL 33136 USA

**Keywords:** Smoking initiation, Family engagement and tobacco use, Parental monitoring and tobacco use, Youth susceptibility to smoke

## Abstract

**Background:**

Family engagement has been shown to play a crucial role in youth cigarette use prevention and uptake. We examine cross-sectional and longitudinal data to determine whether changes in parental monitoring factors influence changes in smoking susceptibility.

**Methods:**

Two cross-sectional surveys of Florida youth (12–17 years) were conducted in 2009, with a follow-up survey in 2010. Multivariable analyses examined demographics, parent characteristics, family engagement, and parental monitoring on youth susceptibility to smoke.

**Results:**

Cross-sectional data show eating together 6+ times/week and doing something for fun 5+ times/week were related to an increased likelihood of Very Low and decreased likelihood of High susceptibility, respectively. Parental monitoring factors and parents tell on a friend who smokes was significantly related to having Very Low susceptibility in both surveys. Mother’s education, parent smokes, family engagement factors, and parental monitoring were significant in both survey rounds. Longitudinal analyses showed change in eating together did not significantly affect the odds of change in smoking susceptibility; however, change in the frequency of doing things for fun with a parent showed decreased odds of susceptibility (OR = .63 [.49–.82]), opposite of the hypothesized direction. Lastly, as youth aged, they were more likely to experience a greater odds of decreased susceptibility (OR_14-15y_ = 1.47 [1.08–1.99] and OR_≥16y_ = 1.40 [1.05–1.84], respectively) and less likely to experience an increased odds of susceptibility (OR_14-15y_ = .65 [.49–.86] and OR_≥16y_ = .72 [.56–.93], respectively).

**Conclusions:**

We found mixed results for family engagement and parental monitoring on changes in youth smoking susceptibility. Cross-sectional data showed general associations in the expected direction; however, longitudinal analyses showed family engagement variables had significance, but in the opposite hypothesized direction.

## Background

Data show youth often participate in activities or risk behaviors that can negatively impact them [1]. Specific to tobacco use, researchers report family engagement can play a crucial role in youth cigarette use prevention and uptake. At the core of this advocacy have been researchers at the National Center on Addiction and Substance Abuse (CASA). CASA conducted annual surveys of youth (12–17 years) from 1999 through 2011, except for year 2000. Their measure of family engagement is the frequency with which youth eat their evening meal together with other family members. Results have been fairly consistent across surveys; youth eating family meals together at least five times per week were less likely to participate in risk behaviors like alcohol consumption, substance use, or tobacco use [2]. Comparatively, youth reporting eating family meals together infrequently (<3 times a week) were about four times more likely to smoke [2]. The authors attribute this to families being engaged; that is, it is not the act of the frequency of eating together, rather it is the family dynamic that happens during dinner. They argue that family togetherness and the conversations that happen over dinner are key factors affecting youth and their relationships with their parents [2, 3]. Further, they examined associations between other variables, such as the amount of time it takes to eat a meal, parental agreement on key issues, time spent together, and the role of older sibling behavior [2, 3]. For instance, youth who spent limited time with their families, measured by how long a family meal lasts and how often they eat together, youth who had parents who did not agree on key issues/messages regarding alcohol and drug use, and youth who reported having declining relationships with their siblings were more likely to report engaging in risk behaviors like alcohol or drug use [2]. Recently, the Nation’s Health reiterated how parents can prevent youth from smoking (and drinking or using drugs) by having their evening meals as a family [4]. Other studies employing different measures of family engagement or parental monitoring show associations with tobacco use of a similar magnitude to those found in the CASA data. For instance, Resnick et al. [5] found parent-family connectedness (e.g., shared activities or parental presence) to be protective for tobacco use. Additionally, parental monitoring (e.g., parents who knew what their children were doing, parents who tell a friend’s parents if the child was smoking, etc.) also have been found to be protective for tobacco use behavior [6–8]. However, previous work has not examined susceptibility items, particularly as they relate to tobacco use.

Many tobacco control programs in the US are based on the Centers for Disease Control and Prevention (CDC) recommended logic model [9]. In short, this model postulates that changes in knowledge and attitudes and beliefs influence susceptibility to use tobacco and that susceptibility also directly influences tobacco use behaviors in youth [9–12]. Therefore, managing susceptibility to smoke is a key factor in any anti-tobacco prevention program [9–12].

The CASA data show an historical sequence of data, exceeding a decade, with surveys conducted as random cross-sectional samples. However, the influence of change in frequency of eating together cannot be linked to change in smoking. Change in either variable is not measured in the CASA data nor is it measured in other studies assessing engagement or parental monitoring. Within this context, we build on previous studies by examining variables that have not been associated in other studies with susceptibility of youth to smoke.

In this paper, we use youth survey data to examine changes in youth susceptibility to smoke, demographic factors, parent characteristics, family engagement, and additional measures of parental monitoring. Specifically, we employ change in youth susceptibility to smoke as the dependent variable; youth anti-tobacco programs attempt to prevent tobacco use initiation through anti-tobacco messaging that achieve low levels of susceptibility to smoke, which is a key step in the prevention process. In other words, youth who have low levels of susceptibility to smoke are less likely to initiate tobacco use [11]. Second, in addition to family engagement items, we employ measures to understand parental monitoring. Third, we include demographic and parent characteristic variables as controls, which also are well established factors influencing susceptibility to smoke [13–15]. Finally, our samples allow us to assess family engagement and parental monitoring in a cross-sectional and longitudinal context to determine whether these factors influence change in susceptibility over time.

## Methods

### Sample

Two cross-sectional telephone surveys were conducted in Florida by the University of Florida Survey Research Center. Survey I was completed in January 2009 and Survey II in August 2009. The sampling frame, created by Genesys, Inc., is a vendor generated list to maximize the probability that calls would target a household containing a youth (12–17 years), from which a random sample of youth was taken [10, 16, 17]. Both cellular and landline telephone numbers were included in the sampling frame. Parent consent was obtained first then the targeted youth’s consent. Youth who completed the survey received $12.50 for participation. The first cross-sectional survey had a final sample size of 2200 and the second 2203 [10, 16]. All samples are representative of youth in Florida based on Census data for gender, race/ethnicity, and school enrollment.

Respondents who completed the surveys also are asked if they are willing to be interviewed in a future research study. Participants responded positively to this item (96 and 95 %, respectively). These respondents then were pooled to form the sampling frame for the follow-up survey completed in May 2010, approximately 17 months and about 11 months after the respective cross-sectional surveys. The follow-up survey was comprised of 1999 youth who again are representative of youth in Florida based on Census data for gender, race/ethnicity, and school enrollment. Survey items are identical across all of the surveys. We allowed up to 10 call-backs per household for each survey round. The project was approved by the University of Miami Miller School of Medicine IRB and the University of Florida.

### Measures

#### Dependent variable

Two dependent susceptibility variables were created. First, a 42-item attitude/belief inventory, plus three additional items (number of best friends who smoke, smoke a cigarette if offered by a best friend, and use/wear an item with a tobacco company logo on it), were included in the factor analysis to create the susceptibility to smoke variable. Items contained a Likert type response scheme, except for one, where respondents indicated a number (coded 0–3 with “0” representing the lowest level of risk). We did a principal components factor analysis using a varimax rotation and cutoff of 1.0. Then we formed summary scores for each factor using the items that had a rotated factor loading greater than or equal to 0.4. We report the chronbach alpha for the scale scores below.

The factor analysis yielded a 10-item factor to assess susceptibility. They are:

1. If someone you thought was cool offered you a cigarette, you would smoke it.
2. There is a lot of peer pressure to smoke.
3. As you get older, you notice that more of your friends are not interested in smoking.
4. You really notice that fewer and fewer people are smoking.
5. You will use a tobacco product like snus, snuff, or chewing tobacco in the next year.
6. Most people your age do not believe all the bad things they hear about tobacco products.
7. You will smoke a cigarette in the next year.
8. You notice that people your age do not want to be around other young people who smoke.
9. If your best friend offered you a cigarette, you would smoke it.
10.  How many of your four best friends smoke cigarettes?

The correlation matrix showed a moderate relationship to one another and a strong tendency for each item to be associated with the total score than with any of the other items. The *chronbach alpha* is .693, suggesting modest internal consistency.

Next, we examined means and standard deviations of the raw susceptibility scores for youth and for youth by age and gender (see left columns of Table 1). As youth age, we would expect their smoking susceptibility levels to increase. Indeed, scores are in the expected direction and the magnitude of the standard deviations relative to the means suggests the distributions are acceptable. Youth scoring one standard deviation or less below the mean (0–4) are considered to have “Very Low” susceptibility, scoring roughly one standard deviation below the mean to the mean (5–8) is “Low” susceptibility, and persons scoring from the mean to one standard deviation above the mean (9–11) have a “Moderate” level of susceptibility. All youth scoring 12 or more are treated as having “High” susceptibility. The susceptibility measure is derived from the first survey data and we performed similar analyses using the second survey data showing similar results, hence we used the same procedures for setting categories for measuring susceptibility in succeeding surveys.

**Table 1:** Distribution of smoking susceptibility levels for all youth and by gender and age group (*n* = 2200)

Group			Very low		Low		Moderate		High	
	Mean	SD	N	%	N	%	N	%	N	%
All youth	8.26	3.84	304	13.8	961	43.7	574	26.1	361	16.4
Gender										
Female	8.20	3.64	134	12.4	485	45.0	290	26.9	168	15.6
Male	8.32	4.04	170	15.1	476	42.4	284	25.3	193	17.2
Age										
<14	7.04	3.07	146	21.8	400	51.5	183	23.6	48	5.9
14–15	8.66	3.78	84	14.5	278	39.9	197	28.3	138	19.2
16>	9.17	4.31	74	12.0	283	40.0	194	26.7	175	23.7

Additionally, gender and age groups also lend preliminary validity to the measure (Table 1). Gender differences are relatively small and the percent of youth scoring Very Low is inversely related to age, varying from nearly 22 % at the youngest age group to about 12 % for the oldest age group, with the converse showing susceptibility increasing with age.

The second dependent variable was created for the longitudinal analyses. We cross-tabulated the susceptibility measures for Time One (Surveys I and II) by Time Two (Follow-up survey) to show change in susceptibility. Change in susceptibility is measured with a three category variable (1 = increase, 2 = decrease, and 3 = no change (*ref*)).

#### Independent variables

The independent measures include demographics, parent characteristics, family engagement, and four parental monitoring variables. The demographic measure for age is categorical (1 = 16 or older, 2 = 14–15 years, and 3 = less than 14 (*ref*)). Gender is categorical with 0 = female; 1 = male (*ref*). Race/ethnicity is measured as African-American/Black, Hispanic, Other, and White (*ref*).

Two measures are included to assess parent characteristics: 1) mother’s level of education (< than high school, high school graduate, some college, and college graduate (*ref*)) and 2) change in parent smoking (0 = decrease in smoking; 1 = increase in smoking; 2 = no change (*ref*)). The change in parent smoking is a computed variable derived from Survey I or II asking youth if their father or mother currently smoke (yes/no) and from the follow up survey where we asked youth again if their father or mother currently smoked. Respondents who indicated that from Survey I or II to the follow up survey their parent no longer smoked, started to smoke, or were current smokers were listed as decrease in smoking, increase in smoking, or no change, respectively. Two indicators of family engagement parallel the CASA measures, they are: 1) on average how many times a week all members of their household eat their evening meal together (less than 3 (*ref*), 3–5, 6 or more) and on average how many times a week youth did something just for fun with one or both of his/her parents (less than 2 (*ref*), 2–4, 5 or more).

Four additional parental monitoring items are asked: how many of their four best friends their parents know well (0–1 (*ref*), 2–3, 4); how many of their four best friend’s parents their parents know well (0–1 (*ref*), 2–3, 4); if they thought their parents would tell a friend’s parents if they saw the friend smoke (no (*ref*), not sure, yes); and if they thought a friend’s parent would tell their parents if they saw the respondent smoke (no (*ref*), not sure, yes). All measures are self-reported items. Detailed discussions of the survey items and validity of these items have been described previously [8].

#### Analyses

We conducted descriptive statistics, bi-variate analyses, and multivariable analysis on the cross-sectional data as a first step examining smoking susceptibility among youth and demographic factors, parent characteristics, family engagement, and parental monitoring. Next, odds ratios and 95 % confidence intervals were estimated with multivariable polytomous regression to assess changes in family engagement and changes in parental monitoring from Time One to Time Two (Follow up) on change in susceptibility from Time One to Time Two, controlling for demographic variables and Time One susceptibility. Variables significant in the bi-variate analyses were included in the full models. All data analyses were performed using SPSS version 17.0 (SPSS, Inc.).

## Results

### Cross-sectional data

Table 2 shows the cross-sectional bi-variate associations between each independent variable and level of susceptibility per survey. These associations show the percentage of youth having Very Low and High levels of susceptibility for each independent variable. In general, the independent variables were in the expected direction. For instance, as age increases the percent of youth having Very Low susceptibility decreases, and as age increases the percent of youth having High susceptibility increases. This pattern holds for all of the parent characteristic measures, family engagement, and parental monitoring variables. We also see consistency in variables between the first and the second surveys, indicating reliability for the measures of the independent variables. For Survey I this pattern holds for six of the eight independent variables (excluding the demographic) and for Survey II it holds for seven of the independent variables.

**Table 2:** Bi-variate associations between youth characteristics and very low and high smoking susceptibility using cross-sectional data

	Survey I (*n* = 2200)			Survey II (*n* = 2203)		
Demographics	% Very low	% High	N	% Very low	% High	N
Age						
<14	21.8	5.9	762	21.1	6.4	731
14–15	14.5	19.2	697	13.3	19.0	804
16>	12.0	23.7	726	10.6	24.2	654
Gender						
Female	14.7	15.2	1077	15.1	17.6	1118
Male	17.8	16.8	1123	14.9	14.9	1085
Race/Ethnicity						
White	15.8	16.9	1815	14.3	16.6	1352
African American	15.6	9.2	109	17.8	12.9	309
Hispanic	13.9	18.3	115	15.0	17.1	467
Other	22.3	10.1	148	15.9	15.9	63
Parent characteristics						
Mother’s education						
College Grad	17.2	13.5	1332	15.3	14.9	1298
Some college	19.2	19.5	365	15.9	16.4	415
HS Grad	12.4	19.2	355	13.0	20.8	355
Less than HS	7.9	28.1	89	7.8	22.1	77
Parent who smokes						
No	19.4	7.6	1733	15.6	14.1	1770
Yes	13.5	25.3	467	12.5	25.4	433
Family engagement						
Avg times eat evening meal together						
<3	12.9	24.3	519	13.4	24.2	491
3–5	15.0	14.4	848	14.1	16.7	842
6>	19.8	12.5	815	19.0	15.9	857
Avg times do something w/ parent just for fun						
<2	16.5	20.1	740	12.6	24.0	641
2–4	15.1	13.3	1814	17.8	17.4	1195
5>	26.2	13.0	262	19.8	8.5	343
Parent monitoring						
No. best friends parents know well						
0–1	15.2	22.7	290	14.7	21.3	272
2	12.3	17.8	472	12.9	21.7	498
3	14.5	16.4	488	11.9	15.4	421
4	19.4	13.0	947	17.2	12.7	992
No. best friends parents parents know well						
0–1	11.9	24.3	573	12.7	24.4	561
2	15.5	17.8	651	12.7	17.7	648
3	17.5	15.0	481	16.5	11.7	401
4	20.9	7.7	492	18.1	10.5	580
Your parents tell on friend						
No	8.7	40.7	241	8.0	38.4	289
Not sure	12.2	23.1	402	11.1	20.7	135
Yes	18.4	10.4	1556	16.4	12.4	1772
Friend’s parents tell on you						
No	11.0	44.8	145	8.8	45.4	194
Not sure	12.5	24.8	407	10.0	27.5	120
Yes	17.7	11.3	1647	15.9	12.6	1886

Next, as a final step examining parent characteristics, family engagement, and parental monitoring cross-sectionally, we used multivariable logistic regression to calculate adjusted odds ratios between each of the independent indicators and Very Low and High susceptibility, taking account of age, gender, and race/ethnicity (Table 3). That is, partial models were run for each independent indictor controlling for age, gender, and race/ethnicity. When controlling for age, gender, and race/ethnicity, some of the relationships between the independent variables and susceptibility measures are altered. The number of independent variables significantly related to Very Low susceptibility is reduced; for example, neither of the parent characteristics is related to Very Low susceptibility in either survey.

**Table 3:** Multi-variable logistic regression partial models showing the adjusted odds ratios and the association between the independent indicators and very low and high susceptibility

	Survey I (*n* = 2200)				Survey II (*n* = 2203)			
	VL		H		VL		H	
	OR	95 % CI	OR	95 % CI	OR	95 % CI	OR	95 % CI
Parent characteristics								
Mother’s education								
College Grad	___		___		___		___	
Some college	1.30	.96/1.76	1.35	.99/1.85	1.09	.80/1.49	1.06	.77/1.44
HS Grad	.73	.51/1.03	1.37	1.19/1.88	.83	.58/1.18	1.50	1.10/2.04
Less than HS	.46	.21/1.00	2.27	1.37/3.75	.44	.19/1.03	1.96	1.69/3.14
Parent smokes								
No	___		___		___		___	
Yes	.76	.57/1.03	2.10	1.62/2.73	.77	.56/1.06	2.08	1.59/2.70
Family engagement								
No. of days eat together								
<3	___		___		___		___	
3–5	1.16	.84/1.61	.51	.38/.67	1.10	.79/1.53	.54	.41/.73
6>	1.54	1.12/2.11	.48	.35/.65	1.17	.85/1.62	.49	.36/.66
No. times do things for fun								
<2	___		___		___		___	
2–4	.79	.60/1.03	.73	.55/.96	1.18	.89/1.57	.52	.41/.67
5>	1.07	.74/1.56	.77	.51/.98	1.51	1.05/2.17	.36	.23/.55
Parent monitoring								
No. of four best friends parents know well								
0–1	___		___		___		___	
2–3	.87	.59/1.26	.67	.48/.94	.84	.57/1.25	.82	.59/1.18
4	1.38	.95/1.99	.49	.35/.70	1.24	.85/1.81	.53	.37/.77
No. four best friend’s parents parents know well								
0–1	______		___		___		___	
2–3	1.44	1.06/1.96	.60	.46/.77	1.07	.78/1.45	.62	.49/.82
4	1.87	1.33/2.65	.31	.21/.46	1.37	.98/1.91	.43	.31/.81
Your parents tell on friend smoking								
No	___		___		___		___	
Not sure	1.36	.78/2.36	.49	.34/.70	1.27	.64/2.53	.47	.29/.77
Yes	2.03	1.24/3.30	.22	.16/.31	1.89	1.30/2.97	.28	.21/.37
Friend’s parents tell on you smoking								
No	___		___		___		___	
Not sure	1.13	.61/2.09	.49	.34/.70	1.02	.46/2.22	.49	.21/.82
Yes	1.51	.86/2.65	.22	.16/.31	1.56	.93/2.64	.22	.16/.30

Table 3 also shows family engagement variables; that is, eating together 6+ times a week and doing something just for fun 5+ times per week were associated with a greater odds of Very Low susceptibility and a lower likelihood of High susceptibility. Eating together 6+ times a week is significant in the first survey round for Very Low susceptibility, but not the second. Only the parental monitoring variable, your parents tell on a friend, is significantly associated with a greater odds of Very Low susceptibility and a lower odds of High susceptibility in both surveys. In short, of the 16 possible odds ratios examining Very Low susceptibility, only four variables from the first and two variables from the second survey are significantly related.

Second, only 14 of the odds ratios linking the independent variables to High susceptibility are significant in the first and second survey. Of the parent characteristics, mother’s education and parent smokes are significant in survey rounds I and II. However, all of the family engagement factors are significant in both surveys for High susceptibility. Additionally, all of the parental monitoring variables are related to High susceptibility in both surveys (save one, number of best friends parents know well in Survey II). Overall, within a cross-sectional context, parent characteristics, family engagement, and parental monitoring factors are associated with High susceptibility.

### Longitudinal data

We begin the longitudinal analysis examining relationships between Time-one status for each of the independent variables and change in susceptibility from Time-one to Time-two (data not shown). Preliminary data showed race/ethnicity displayed no significance in any of the analyses, therefore, we dropped it from further analysis. Preliminary data analyses did not directly address if changes in family engagement and parental monitoring affect changes in susceptibility. Therefore, using polytomous regression, we ran the full model taking into consideration the demographic variables and the key variables of interest.

We examined changes in family engagement and parental monitoring factors on changes in susceptibility from Time-one to the follow-up survey, with adjusted odds ratios controlling for demographics, parent characteristics, and Time-one level of susceptibility (Table 4). If these changes operate on changes in susceptibility in the hypothesized manner, we expect youth who increase their frequency of family engagement and monitoring between the first and the follow-up survey less likely to have increased odds of susceptibility and more likely to have decreased odds of susceptibility. We found youth with a decrease in family engagement (do something just for fun) had a lower likelihood of a decrease in susceptibility. For five of the six measures assessing change in family engagement and parental monitoring on change in susceptibility, the data suggest there are no significant effects (Table 4).

**Table 4:** Polytomous regression full model adjusted odds ratios for changes in family engagement and parental monitoring on change in smoking susceptibility^a^

	Increase			Decrease		
	OR	95 % CI	*P* value	OR	95 % CI	*P* value
Age						
<14	___	___	___	___	___	___
14–15	1.47	1.08/1.99	.014	.65	.49/.86	.003
16>	1.40	1.05/1.84	.021	.72	.56/.93	.011
Change in family engagement						
Eating together						
No change	___	___	___	___	___	___
Decrease	1.09	.83/1.43	.539	1.05	.81/1.37	.698
Increase	1.13	.81/1.58	.475	1.08	.78/1.48	.656
Do something w/ parent just for fun						
No change	___	___	___	___	___	___
Decrease	.63	.49/.82	.001	.99	.79/1.27	.980
Increase	.81	.58/1.13	.210	.95	.70/1.31	.757
Change in parent monitoring						
Four best friends parents know well						
No change	___	___	___	___	___	___
Decrease	1.30	.99/1.71	.058	1.12	.86/1.44	.405
Increase	1.06	.75/1.49	.748	.79	.56/1.13	.195
Four best friend’s parents your parents know well						
No change	___	___	___	___	___	___
Decrease	.82	.64/1.07	.143	1.06	.83/1.35	.643
Increase	1.11	.77/1.61	.580	.92	.63/1.34	.664
Your parents tell on friend						
No change	___	___	___	___	___	___
No to Yes	1.05	.72/1.51	.773	1.06	.75/1.49	.744
Yes to No	1.08	.65/1.79	.780	.74	.43/1.28	.283
Friend’s parents tell on you						
No change	___	___	___	___	___	___
No to Yes	.83	.56/1.22	.333	1.13	.79/1.61	.517
Yes to No	.84	.43/1.64	.601	.72	.36/1.45	.357

^a^Adjusted for gender, age, parent characteristics, and time one susceptibility

Family engagement and parental monitoring variables do not confirm either of the predictions. In fact, data show no significant change effects for eating together and change in susceptibility to smoke. With respect to change in the frequency of doing things just for fun with a parent, data show a decrease in frequency is associated with lower odds of decreased susceptibility compared to no change (OR = .63 [.49–.82]). With respect to parental monitoring, there are no statistically significant effects on change in susceptibility for changes in the number of four best friend’s parents your parents know well, your parents tell on a friend if they smoked, and friend’s parents tell on you if you smoked. For the number of four best friends parents know well, the data approached statistical significance. Youth who had decreases in the number of friends’ parents know well were more likely to have a greater likelihood of decreased susceptibility (OR = 1.30 [.99–1.71]), again in the opposite hypothesized direction. Finally, age was associated with change in susceptibility from Time-One to Time-Two (Follow up). As youth aged, they were more likely to experience a greater likelihood of decreased susceptibility (OR = 1.47_14-15y_ [1.08–1.99] and OR = 1.40_≥16y_ [1.05–1.84], respectively) and less likely to experience a greater likelihood of increased odds in susceptibility (OR = .65_14-15y_ [.49–.86] and OR = .72_≥16y_ [.56–.93], respectively).

## Discussion

Our primary objective was to determine if changes in family engagement and parental monitoring lead to changes in youth susceptibility to smoke. We first tested this hypothesis employing data from two cross-sectional random samples of Florida youth (12–17 years). We used similar items as the CASA team and included additional measures of parental monitoring, demographic, and parent characteristic variables as controls. Unlike CASA, our samples came from a single State (Florida) and our dependent variable was a 10-item susceptibility scale, not actual cigarette use.

Bi-variate analysis results were similar to those previously reported. For example, the number of times a youth eats together with his/her family per week was inversely related to the likelihood s/he would have High susceptibility to smoke and positively related to the likelihood s/hey will have Very Low susceptibility. Data also showed each of these patterns prevailed for each of the other measures of parental monitoring.

Multi-variable cross-sectional analyses employed adjusted odds ratios and showed the associations between family engagement and parental monitoring factors had decreased odds of High levels of susceptibility, even after adjusting for controls. Additionally, there were significant differences between the frequency of eating the family meal together and the increased odds of having Very Low susceptibility and decreased odds of High susceptibility; this also was evident when examining the frequency of doing things just for fun with a parent and the number of best friends a parent knows well. There were, however, two parental monitoring factors significantly associated with Very Low levels of susceptibility in each survey. Items asking the number of best friends’ parents a youth’s parents knew well and if youth thought their parents would tell on one of their friends if they saw them smoke showed youth were more likely to have Very Low levels of susceptibility.

Next, longitudinal data allowed us to assess changes in family engagement and parental monitoring and changes in susceptibility. We found no evidence to support our hypothesis that changes in family engagement items and parent monitoring items had any impact on changes in susceptibility, after controlling for demographics, parent characteristics, and Time-one level of susceptibility. Yet, one family engagement variable (do something fun with parents) had a decreased odds of susceptibility. Further, one parental monitoring variable approached significance, but this was in the opposite direction of our stated hypothesis. Therefore, we found limited evidence for their role in impacting the risk for cigarette uptake among youth. It appears that only changes in age had an effect on changes in youth susceptibility to smoke, and in the opposite hypothesized direction.

A couple of factors may be responsible for the lack of findings with the longitudinal data. First, as shown in Tables 1, 2, and 3, youth in this study were more likely to have High levels of susceptibility to cigarette uptake (e.g., Table 1 shows 42.9 % of youth 14 and older were considered highly susceptible). Therefore, youth starting with High susceptibility were not in a position to have increased susceptibility in the longitudinal analyses; if there was going to be a change in susceptibility over time, it could only be a decrease. Second, as youth age and become more independent, it is reasonable to presume that parental monitoring decreases. These two factors together may explain why the longitudinal data showed a decrease in family engagement and parental monitoring correlated with a decrease in changes in susceptibility to smoke. Further, the association in changes in susceptibility in the opposite hypothesized direction may be a reflection of a general normative trend happening in Florida; that is, Florida has become increasingly tobacco free and this overall normative trend may be driving these results. Finally, when examining Table 5, we also see that the majority of youth did not change in their susceptibility from Time 1 to the Follow up, rather they stayed the same. It may be that the longitudinal analysis is reflecting this since no change was the reference group in the analyses.

**Table 5:** Change in susceptibility from time-one to follow-up by independent variables (*n* = 1999)

	Change in susceptibility T^1^ – T^2^					
	Decrease		No change		Increase	
T^1^ independent variable status	N	%	N	%	N	%
Demographics						
Age						
<14	127	19.1	320	44.5	262	36.4
14–15	192	27.2	331	46.9	182	25.9
16>	158	27.9	271	47.8	138	24.3
Gender						
Female	255	25.3	494	49.0	259	25.7
Male	235	23.7	432	43.6	324	32.7
Race/Ethnicity						
White	346	23.9	699	48.2	404	27.9
African American	46	26.4	69	39.7	59	33.9
Hispanic	70	29.5	95	40.2	72	30.4
Other	25	20.2	54	43.5	45	36.3
Parent characteristics						
Mother’s education						
College Grad	321	25.5	582	46.3	255	28.2
Some college	77	22.4	147	42.9	119	34.7
HS Grad	62	21.8	151	53.2	71	25.0
Less than HS	18	29.0	24	38.7	20	32.3
Parent smokes						
No	447	24.2	863	46.7	536	29.0
Yes	43	28.1	63	41.2	47	30.7
Family engagement						
No. of days eat together						
<3	116	25.6	212	46.8	125	27.6
3–5	158	21.7	338	46.4	233	32.0
6>	213	26.6	370	46.2	218	27.2
No. times do things for fun						
<2	151	23.9	298	47.2	182	28.8
2–4	273	25.3	506	46.9	299	27.7
5>	64	24.1	111	41.7	91	34.2
Parent monitoring						
No. of four best friends parents know well						
0–1	88	27.4	156	48.6	77	24.0
2–3	226	23.3	339	46.2	296	30.5
4	176	25.0	319	45.3	209	29.7
No. four best friend’s parents parents know well						
0–1	33	20.8	79	49.7	47	29.6
2–3	194	25.8	347	46.1	211	28.1
4	263	24.2	498	45.9	324	29.9
Your parents tell on friend smoking						
No	432	24.1	822	45.9	535	29.9
Not sure	57	27.8	101	49.3	47	22.9
Yes						
Friend’s parents tell on you smoking						
No/Not sure	453	24.4	846	45.6	556	30.0
Yes	37	26.2	78	55.3	36	18.4

It is important to view these findings with several limitations in mind. First, while our dependent variable of susceptibility was created from a 10-item factor, with five of the variables having known validity and reliability [16, 18-20], we did not take into consideration youth smoking status (ever try or current); therefore, future research should address this constraint and take into consideration youth smoking status to fully understand youth smoking patterns and susceptibility to smoke. In addition, it is customary to report a response rate when conducting survey research. However, for this study there are no reportable response rates since the survey provider, the University of Florida Survey Research Center, no longer calculates response rates. It should be noted though that in general telephone survey response rates tend to be low. Finally, our measure of susceptibility is a categorical variable and may not fully reflect subtle changes in susceptibility over time. Therefore, this may constrain the analyses. With these limitations in mind, we can draw attention to some strong points of the paper.

Whereas the main hypotheses were not born out, this paper contains several strengths. First, previous studies examining tobacco use in youth and the role of parental engagement and parental monitoring have used cross-sectional analyses. Longitudinal data have not been used. When using cross-sectional data, assumptions may be made about changes in behavior; however, only longitudinal data can speak directly about individual behavior change. In this case, the longitudinal data do not show changes toward increased susceptibility. To highlight these differences, we use both the cross-sectional and longitudinal data to show changes in youth behaviors as they relate to changes in susceptibility to smoke and this strengthens the results of the paper. Overall, we used a random sample of youth for our data and multiple measures of parental engagement and parental monitoring, which are all strengths of the paper, to examine the relationship between changes in behavior and changes in susceptibility.

## Conclusions

In sum, we found mixed results for the effects of family engagement and parental monitoring on changes in youth susceptibility to smoke. Cross-sectional data showed a general association in the expected direction between youth susceptibility and the independent variables. The cross-sectional data also show not all of the adjusted odds ratios were significant. Finally, we examined changes in youth susceptibility to smoke in the longitudinal survey and found one family engagement variable had significance, but in the opposite hypothesized direction.

## Abbreviations

CASA: National Center on Addiction and Substance Abuse
CDC: Centers for Disease Control and Prevention
